# Adverse respiratory patterns in near-term spontaneously breathing newborn lambs with elevated airway liquid volumes at birth

**DOI:** 10.3389/fped.2024.1336154

**Published:** 2024-04-16

**Authors:** I. M. Davies, K. J. Crossley, E. V. McGillick, I. Nitsos, K. Rodgers, A. Thiel, V. A. Zahra, S. Badurdeen, A. B. te Pas, S. B. Hooper

**Affiliations:** ^1^ The Ritchie Centre, Hudson Institute of Medical Research, Melbourne, VIC, Australia; ^2^ Department of Obstetrics and Gynaecology, Monash University, Melbourne, VIC, Australia; ^3^ Department of Paediatrics, Mercy Hospital for Women, Heidelberg, VIC, Australia; ^4^ Neonatal Research, Murdoch Children’s Research Institute, Melbourne, VIC, Australia; ^5^ Division of Neonatology, Department of Pediatrics, Leiden University Medical Center, Leiden, Netherlands

**Keywords:** respiratory distress, airway liquid volume, cardiorespiratory transition, breathing patterns, respiratory function, newborn

## Abstract

**Introduction:**

Recent evidence indicates that respiratory distress (RD) in near-term infants is caused by elevated airway liquid (EL) volume at the beginning of air-breathing after birth. While the adverse effects EL volumes on newborn lung function are known, the effects on respiratory control and breathing patterns shortly after birth (<4 h) are unknown. We investigated the effects of EL volumes on cardiorespiratory function and breathing patterns in spontaneously breathing near-term newborn lambs in the first hours after birth.

**Methods:**

At 137–8 days gestation (2–3 days prior to delivery; term ∼147 days), sterile surgery was performed on fetal sheep (*n* = 17) to implant catheters and blood flow probes. At 140 days, lambs were delivered via caesarean section under spinal anaesthesia. Airway liquid volumes were adjusted to mimic the level expected following vaginal delivery (∼10 ml/kg; Controls; *n* = 7), or elective caesarean section (∼30 ml/kg; elevated airway liquid group; EL; *n* = 10). Spontaneous breathing and cardiorespiratory parameters were recorded over four hours after birth. Non-invasive respiratory support with supplemental oxygen was provided if required.

**Results:**

EL lambs required higher inspired oxygen levels (*p *= 0.0002), were less active (*p *= 0.026), fed less (*p *= 0.008) and had higher respiratory morbidity scores than Controls (*p *< 0.0001). EL lambs also displayed higher rates of breathing patterns associated with RD, such as expiratory braking and tachypnoea. These patterns were particularly evident in male EL lambs who displayed higher levels of severe respiratory morbidity (e.g., expiratory braking) than female EL lambs.

**Conclusion:**

The study demonstrates that EL volumes at birth trigger respiratory behaviour and breathing patterns that resemble clinically recognised features of RD in term infants.

## 1 Introduction

Neonatal respiratory distress (RD) is common in preterm infants, as they have structurally immature lungs that are surfactant deficient and difficult to aerate. However, significant numbers of infants born at or near-term (>37 weeks gestation) also experience RD, despite these infants having mature lungs and being otherwise healthy at birth. These infants often require admission into intensive care shortly after birth for breathing support, which separates them from their parents and increases their risk of morbidities such as pulmonary hypertension (1). In near-term infants, RD is characterised by rapid, shallow breathing (tachypnoea), expiratory braking manoeuvres, hypoxemia, chest retractions, and nasal flaring (2). However, as these symptoms are non-specific and the mix of symptoms and severity of each can vary between infants, the underlying cause of RD at or near-term has remained poorly understood. In addition, as term infants without RD can display similar characteristics, the diagnosis of term RD is subjective and early warning signs can be missed.

The mode of delivery is a major contributing risk factor for developing RD in near-term infants, with caesarean section (CS) without labour carrying a 2–4 fold higher risk compared to vaginal birth (3–6). Before birth, the lungs are liquid-filled and at birth this liquid must be cleared to allow the entry of air and the onset of pulmonary gas exchange (7). While historically the precise timing for airway liquid clearance around or at birth has been a subject of debate, in the absence of any underlying issues (e.g., loss of amniotic fluid), it is now clear that lung liquid volumes remain high until the onset of labour (35–40 ml/kg) (8). Uterine contractions during labour and vaginal birth increase fetal spinal flexion, which increases intrathoracic pressure due to upwards displacement of the diaphragm (9), causing significant amounts of airway liquid loss via the nose and mouth prior to delivery (7). As such, only relatively small volumes of liquid need to be cleared to establish air-breathing after birth (10); residual airway liquid volumes are expected to be ∼10–15 ml/kg after vaginal delivery (8). In contrast, infants delivered by elective CS without labour at or near-term, are not exposed to these postural changes and, therefore, the volume of airway liquid they must clear after birth is considerably greater and similar to the volume present in the airways in late gestation (8).

As all liquid present in the airways at the onset of breathing must be cleared into the lung parenchyma (11), the presence of larger airway liquid volumes at birth exacerbates the degree of pulmonary oedema and the increase in lung interstitial tissue pressures that normally occurs after birth (12). Recent evidence indicates that this high degree of pulmonary oedema after birth is responsible for the RD observed in term infants (12–14). In comparison to airway liquid volumes expected following vaginal delivery, when volumes remain at levels expected in late gestation fetuses prior to labour onset (i.e., elective CS with no labour), this causes: (i) chest wall expansion, (ii) flattening of the diaphragm, (iii) a decrease in lung compliance, (iv) a decrease in end-expiratory lung volume (functional residual capacity; FRC), (v) a reduction in pulmonary blood flow and (vi) a markedly reduced capacity for oxygen exchange in the immediate newborn period (12, 13, 15). However, the effect of elevated airway liquid volume at birth on respiratory control and breathing patterns in the early newborn period are currently unknown.

It is understood that respiratory centres within the brainstem that control breathing activity are influenced by factors such as hypoxemia and carbon dioxide (16), exposure to cold stimuli (17), as well as endocrine factors such as adenosine, prostaglandins and caffeine (via adenosine receptor) (18). In adults, pulmonary oedema is known to stimulate tachypnoea (19), which is mediated by activation of pulmonary “J receptors” that signal via afferent nerves fibres running within the vagus (19). However, the effect of pulmonary oedema on respiratory control in the newborn is unknown. We propose that breathing patterns observed in term infants with RD may be due to excessive pulmonary oedema caused by the presence of larger than normal volumes of airway liquid at birth.

The aim of this study was to investigate how the volume of airway liquid present at birth influences breathing patterns and respiratory behaviour in spontaneously breathing near-term newborn lambs. We compared airway liquid volumes expected in near-term fetuses delivered by caesarean section without labour (∼30 ml/kg) with the volumes expected in infants following vaginal birth (∼10 ml/kg). We hypothesised that higher airway liquid volumes would adversely affect oxygen exchange and increase the incidence of breathing patterns commonly associated with RD, such as expiratory braking, grunting and tachypnoea. From our observations, we also identified the unanticipated finding that males and females responded differently to EL at birth, thus an investigation into sex-related differences was conducted as a secondary aim.

## 2 Materials and methods

### 2.1 Ethical approval

All experimental procedures were approved by the Monash Medical Centre Animal Ethics Committee and Monash University (MMCA2020/01). All experiments were conducted in accordance with the National Health and Medical Research Council (NHMRC) of Australia code of practice for the care and use of animals for scientific purposes (20). Methodological reporting is provided as per the relevant ARRIVE guidelines (21). Data from Control lambs in this study have been reported previously and were used to characterise the normal respiratory patterns displayed by lambs in the immediate newborn period (22).

### 2.2 Fetal instrumentation

At 126 days gestation (term ∼147 days), pregnant Merino X Border-Leicester ewes were administered medroxyprogesterone acetate (150 mg; i.m. injection) to prevent spontaneous labour (*n* = 17). At 137–8 days gestation, ewes underwent sterile surgery to instrument the fetus with catheters and flow probes as previously described (22). Briefly, under general anaesthesia the fetal head and chest were exposed via hysterotomy to implant vascular catheters into the carotid artery (for blood sampling) and jugular vein. Ultrasonic blood flow probes (Transonic Systems, Ithaca, New York, USA) were placed on the left pulmonary artery and the right common carotid artery. A balloon tipped catheter was also inserted into intrapleural space and a small polyvinyl catheter (2.6 mm O.D.) was inserted into the trachea to measure tracheal pressure, leaving the tip approximately 3 cm below the larynx (23). Following instrumentation, the fetus was returned to the uterus before it and the maternal abdomen were sutured closed; catheters and flow probes were exteriorised via the ewe's flank. Antibiotics were administered daily for three days post-operatively (Cefazolin, AFT Pharmaceuticals; maternal i.v. 1,000 mg; fetal i.v. 100 mg; intra-amniotic 400 mg) and ewes received analgesia via transdermal fentanyl patch (75 μg/h).

### 2.3 Caesarean section delivery under spinal anaesthesia

Near-term, at 140 days gestation, lambs were randomised into one of two groups (Control vs. elevated airway liquid; EL). Amniotic fluid volumes were passively reduced (∼2 h prior to delivery) to simulate membrane rupture before lambs were delivered via CS under spinal anaesthesia as previously described (22). Ewes were briefly (3–10 min) sedated (Propofol 1%; 20–40 mg bolus; i.v.) to inject the spinal anaesthesia (Lignocaine 2%; 0.1 ml/kg) and induce a complete spinal block of the lower body. Following recovery from propofol, ewes were lightly sedated (Midazolam; 1 mg/kg/h; i.v. infusion) and given oxygen via nasal prongs (100% O_2_, flow rate 5 L/min). Immediately prior to delivery, fetal lung liquid was withdrawn directly from the tracheal catheter to reduce airway liquid volumes to levels expected following vaginal birth (∼10 ml/kg). Control lambs [*n* = 7 (22);], had no liquid returned to their airways, whereas lambs with EL (*n* = 10), had a fixed volume of liquid (20 ml/kg estimated body weight Hartmann's solution) added back to the airways via the tracheal catheter to restore airway liquid volumes to a level expected prior to labour onset (8). Hartmann's solution was chosen to replace airway liquid as it is osmotically similar to endogenous fetal lung liquid (24). Control lambs were expected to have ∼10 ml/kg in their airways following delivery, whereas EL lambs were expected to have ∼30 ml/kg following delivery, representing what we expected to be a mild form of RD.

Lambs were delivered, fitted with nasal prongs to give oxygen (gas flow rate 10 L/min; 100% O_2_) and were physically stimulated to encourage spontaneous breathing. Lambs were administered with Flumazenil (0.01 mg/kg i.v.) and Naloxone (0.01 mg/kg; i.v.) to block any depressive effects that maternally administered midazolam and fentanyl could have on respiratory patterns. In addition to physical stimulation, caffeine (20 mg/kg; i.v. bolus) was given to stimulate breathing (a second dose was administered if required), but only during the immediate transitionary period (first 10 min). If the lamb did not establish regular stable breathing or had poor respiratory efforts, a gradually increasing level of respiratory support was provided. This commenced with continuous positive airway pressure (CPAP) provided by via nasal prongs and could be escalated to (i) intermittent positive pressure ventilation (iPPV) via a laryngeal mask (LMA; v-gel® advanced size R6, Docsinnovent Ltd., UK) and if required (ii) intubation (MD03345-4.0, cuffed, Portex Ltd, England) and iPPV, while still on the umbilical cord, with a peak inflation pressure (PIP) of 35 cmH_2_O and a positive end expiratory pressure (PEEP) of 5 cmH_2_O. If intubated, lambs were briefly ventilated in volume-guarantee assist control mode and then switched to CPAP via the ET tube as soon as spontaneous breathing commenced. Once regular breathing was achieved, the umbilical cord was clamped and lambs were transferred to a custom-made sling under a radiant heater. All intubated lambs were extubated within the first 10 min after umbilical cord clamping.

### 2.4 Postnatal lamb monitoring

Physiological recordings of breathing movements, arterial blood flows (pulmonary and carotid) and pressures were measured and recorded electronically (Powerlab, ADInstruments, Castle Hill, Australia) for four hours after birth. Arterial blood samples (0.3 ml) for blood gas analysis were collected every 5 min for the first 30 min after umbilical cord clamping, then every 10 min up until one hour and then every 20 min thereafter until four hours after birth. Oxygen supplementation was provided via nasal prongs if required (gas flow rate 0–15 L/min) to maintain arterial oxygen saturations (SaO_2_) > 90%. Lamb core body temperatures were measured continuously via rectal temperature probe and were maintained at ∼39 °C. Lambs received regular enteral feeds with formula (Profelac Shepard milk replacement; ProviCo Nutrition; Victoria, Australia) with volume intake recorded. Additional fluid was given as an infusion (glucose 5%, 6 ml/kg/h, i.v.). Urine output was measured by recording nappy weight in hourly increments after birth. Environmental stimuli (bright lights, loud noises, physical touching of the lamb) were minimised to avoid arousing the lamb and disturbing the breathing and other physiological recordings. All animals survived until 4 h after birth.

### 2.5 Post-mortem analysis

Ewes were euthanised immediately after umbilical cord clamping and lambs were euthanised at four hours after delivery with an i.v. overdose of sodium pentobarbitone (>100 mg/kg; Virbac Pty. Ltd., Peakhurst, Australia). Lamb body and organ weights were recorded. The bladder was drained of urine and the volume added to the volumes measured from the total nappy weight to generate a cumulative urine output. The lungs were removed and total wet lung weight recorded before a section of lung tissue was taken from both left (upper and lower) and right (upper, middle, and lower) lobes to determine wet/dry weight ratios as previously described (25).

### 2.6 Analysis of physiological recordings and breathing patterns

Physiological measures (20 s epochs) of pulmonary and carotid artery blood flow, arterial blood pressure, heart rate, and respiratory rate were analysed to coincide with the timing of arterial blood gas samples. Alveolar-arterial differences in oxygen (AaDO_2_) values were calculated from physiological measures as previously described (12).

Breathing recordings were deidentified and every minute of breathing recording was examined (by IMD) and categorised into one of seven breathing patterns: (i) quiet, (ii) breathing while active/moving, (iii) breathing during oral feeds, (iv) tachypnoea (>70 breaths/min), (v) expiratory pauses or expiratory holds, (vi) expiratory braking manoeuvres (grunting and diaphragmatic braking), and (vii) step changes in ventilation. Definitions of the breathing pattern classification system and examples of breathing patterns were previously described in (22). To be counted as one of these defined breathing patterns, the duration of the displayed pattern had to be ≥30 s. Additional isolated breathing events including sighs and post-inspiratory diaphragmatic contractions (observed as “double breaths”) were manually counted.

### 2.7 Morbidity scoring

As the type and intensity of respiratory patterns that were indicative of RD varied between lambs, we developed a morbidity score to integrate the adverse breathing patterns in order to quantify the degree of RD between groups. These morbidity scores were based on the criteria outlined in Table 1, with the minimum measures required to calculate a score being: (i) mode of respiratory support, (ii) arterial blood sample, (iii) oxygen requirement (fraction of inspired oxygen; FiO_2_), (iv) mean respiratory rate, (v) categorisation of breathing patterns (blinded), and (vi) visual confirmation of physical signs of distress (noted during experiment). Two animals (1x Control and 1x EL) did not have arterial samples available after 80 min of the experiment and so did not contribute to the morbidity scores during this period.

**TABLE 1:** Lamb respiratory morbidity scoring system.

Measure	Morbidity Score			
	0	1	2	3
Respiratory support requirements	Not required *or* Gas flow ≤5 L/min *(nasal cannula)*	Gas flow 5–15 L/min *(nasal cannula)*	CPAP *(laryngeal mask or ET tube)*	Mechanical Ventilation *(ET tube)*
Acidosis (pH)	7.35–7.45	7.25–7.34	7.15–7.24	<7.15
Fraction of inspired oxygen (FiO_2_, %)	21 (Room air)	22–49	50–79	80–100
Respiratory Rate (breaths/min)	40–69	70–99	100–149	>150
Expiratory Braking	Absent	Events present (<30 s in length)	Events present (≥30 s in length)	
Expiratory Pause or Holding	Absent	Events present (<30 s in length)	Events present (≥30 s in length)	
Isolated breathing events *(Post-inspiratory diaphragmatic contractions, apneas)*	Absent	1 sign present	2 signs present	
Physical signs of distress *(Nasal flaring, intercostal retractions, abdominal efforts, mouth breathing, audible congestion)*	Absent	1 sign present	2 signs present	3 or more signs present
			Total score	/21

CPAP, continuous positive airway pressure; ET, endotracheal tube.

### 2.8 Statistical analysis

Statistical analysis was performed using Prism v9 (Graphpad Software, San Diego, CA) and *p *≤ 0.05 was considered statistically significant. All data were tested for normality (Shapiro-Wilk normality test), transformed where appropriate and are represented as mean ± standard error of the mean (SEM). Lamb sex was analysed using Fisher's exact test for categorical variables. Gestational age, fetal blood gas values, body/organ weights, lung liquid volumes, milk consumption and urine output were analysed using an unpaired Student's *t*-test (Control vs. EL). Post-delivery blood gas values, continuous physiological measures, proportion of time spent in each breathing pattern, isolated breathing events and morbidity scores were analysed by mixed-effects model for treatment (Control vs. EL and EL males vs. EL females), time and interaction followed by Sidak's *post hoc* analysis for multiple comparisons at individual timepoints. Survival curves were analysed using Log-rank (Mantel-Cox) test. Total proportion of time spent in each breathing pattern or total number of isolated breathing events were compared between Control, EL males and EL females using One-way ANOVA with Tukey's *post hoc* analysis for multiple comparisons. A forward stepwise multiple linear regression was used to predict the effect of various respiratory features and presence of EL on gas exchange capabilities (AaDO_2_) from 30 min after umbilical cord clamping until the end of the experiment. As there was a significant interaction of sex on the outcome variable (*p *< 0.0001), regression models were run in males and females separately. *F*-test model comparison was used to distinguish between a set of possible models describing the relationship between time after birth, pH status after birth, presence of EL, respiratory patterns (expiratory braking), respiratory rate and isolated respiratory features (sighs and post-inspiratory diaphragmatic contractions) with AaDO_2_ in either male or female lambs.

## 3 Results

### 3.1 Fetal baseline and lamb characteristics

Gestational age, sex and fetal blood gas values were similar in Control and EL lambs (Table 2). The time taken to establish consistent breathing was similar in Control and EL lambs, with most lambs establishing breathing with nasal oxygen only (5/7 Controls and 6/10 ELs; Table 2). During the experimental period, EL lambs consumed less milk than Control lambs (230 ± 39.5 ml vs. 98.0 ± 21.7 ml, *p *= 0.008, Table 2), although urine output tended to be increased in EL lambs (*p *= 0.057, Table 2). Wet/dry lung weight ratios at post-mortem (∼4 h after birth) were similar in Control and EL lambs (Table 2).

**Table 2:** Lamb baseline characteristics before and after delivery including fetal blood gas values and respiratory support provided during resuscitation on the umbilical cord.

Measure	Control (*n* = 7)	Elevated liquid (*n* = 10)
Gestational age at delivery (days)	140 ± 0	140 ± 0
Body weight (kg)	4.90 ± 0.16	4.05 ± 0.30*
Male: female	4:3	5:5
Fetal blood gas values		
*pH*	7.40 ± 0.0	7.38 ± 0.0
*pCO_2_ (mmHg)*	49.5 ± 1.7	47.7 ± 2.0
*pO_2_ (mmHg)*	18.6 ± 1.2	17.4 ± 1.7
*SaO_2_ (%)*	53.4 ± 3.7	50.0 ± 5.6
*Lactate (mmol/L)*	1.80 ± 0.3	1.83 ± 0.1
*Acid-Base Excess (mmol/L)*	6.02 ± 1.0	3.04 ± 1.1
Prior to delivery		
Amniotic fluid drained (mL)	507 ± 145	322 ± 95
Fetal lung liquid drained from trachea (mL/kg)	8.13 ± 2.46	8.66 ± 1.89
Liquid returned to the airways (Hartmann's solution; mL/kg)	N/A	22.86 ± 0.93
Maximum respiratory support required during resuscitation on umbilical cord (stepwise escalation)		
*None*	1/7	0/10
*Nasal Oxygen*	5/7	6/10
*Laryngeal mask + CPAP/ iPPV*	0/7	0/10
*Endotracheal intubation + iPPV*	1/7	4/10
Time from first breath to umbilical cord clamping (mins)	6.64 ± 1.67	10.06 ± 2.53
Postmortem		
Milk consumption after delivery (mL)	230.0 ± 39.5	98.0 ± 21.7*
Urine output after delivery (mL)	165 ± 28	254 ± 31
Wet/dry lung weight ratio (g/g)	5.71 ± 0.15	6.82 ± 0.46

Data are mean ± SEM. Sex was analysed as a categorical variable using Fisher's exact test. Respiratory support methods were compared using Chi square test for trend. All other measurements were analysed by unpaired Student's *t*-test. *p ≤ 0.05. CPAP, continuous positive airway pressure, iPPV, intermittent positive pressure ventilation.

### 3.2 Effect of EL on oxygenation and blood gas status immediately after birth

**Figure 1:**
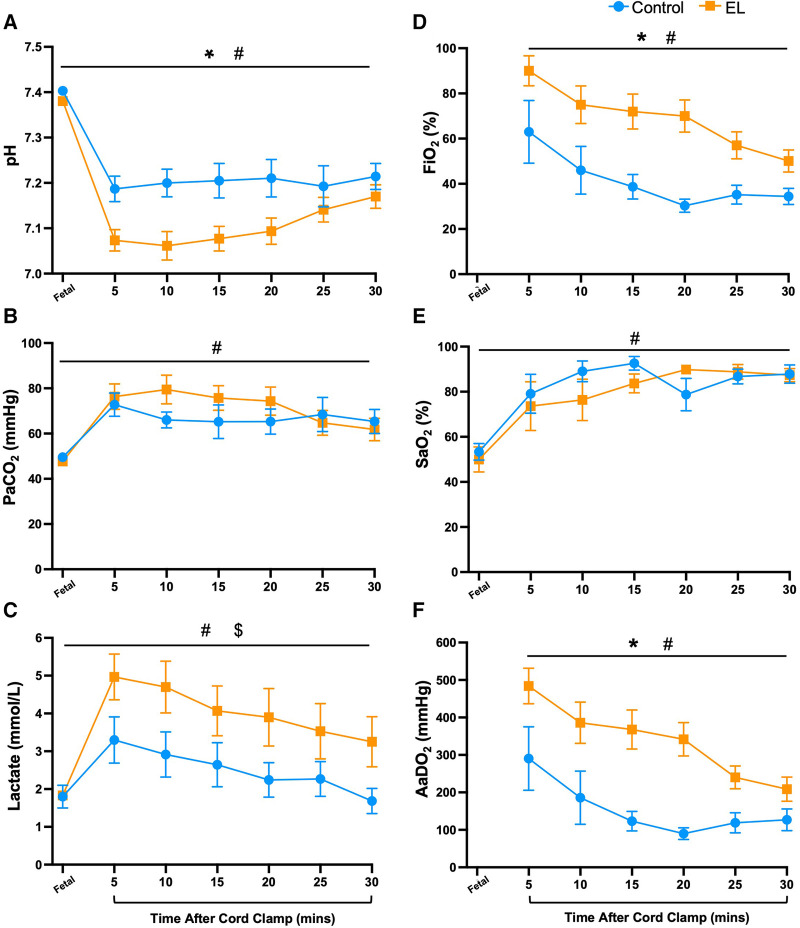
Blood gas status and oxygen requirements immediately after birth in control and EL lambs. Arterial pH (**A**), partial pressure of carbon dioxide (PaCO_2_, **B**) and lactate (**C**), fraction of inspired oxygen (FiO_2_; **D**) arterial oxygen saturation (SaO_2_; **E**), and alveolar-arterial difference in oxygen (AaDO_2_; **F**) over the first 30 min after umbilical cord clamping in Control (blue circles; *n* = 7) and elevated liquid (EL; orange squares; *n* = 10) lambs. Data are mean ± SEM. Mixed effects analysis: *p ≤ 0.05 = effect of treatment. ^#^*p *≤ 0.01 = effect of time, ^$^*p *≤ 0.05 = interaction effect (treatment × time).

In the first 30 min after umbilical cord clamping, EL lambs had lower arterial pH levels (*p *= 0.019, Figure 1A) and tended to have higher partial pressure of carbon dioxide (PaCO_2_) levels than Controls, although the latter were not statistically different (*p *= 0.47, Figure 1B). EL lambs required more supplemental oxygen (FiO_2_; *p *= 0.0004, Figure 1D) and had significantly higher alveolar-arterial differences in oxygen (AaDO_2_) than Controls, reflecting poorer gas exchange (*p *= 0.0002, Figure 1F). However, both groups demonstrated an improvement in gas exchange over time (*p *< 0.0001, time effect, Figure 1F).

### 3.3 Effect of EL on respiratory behaviour immediately after birth

**Figure 2:**
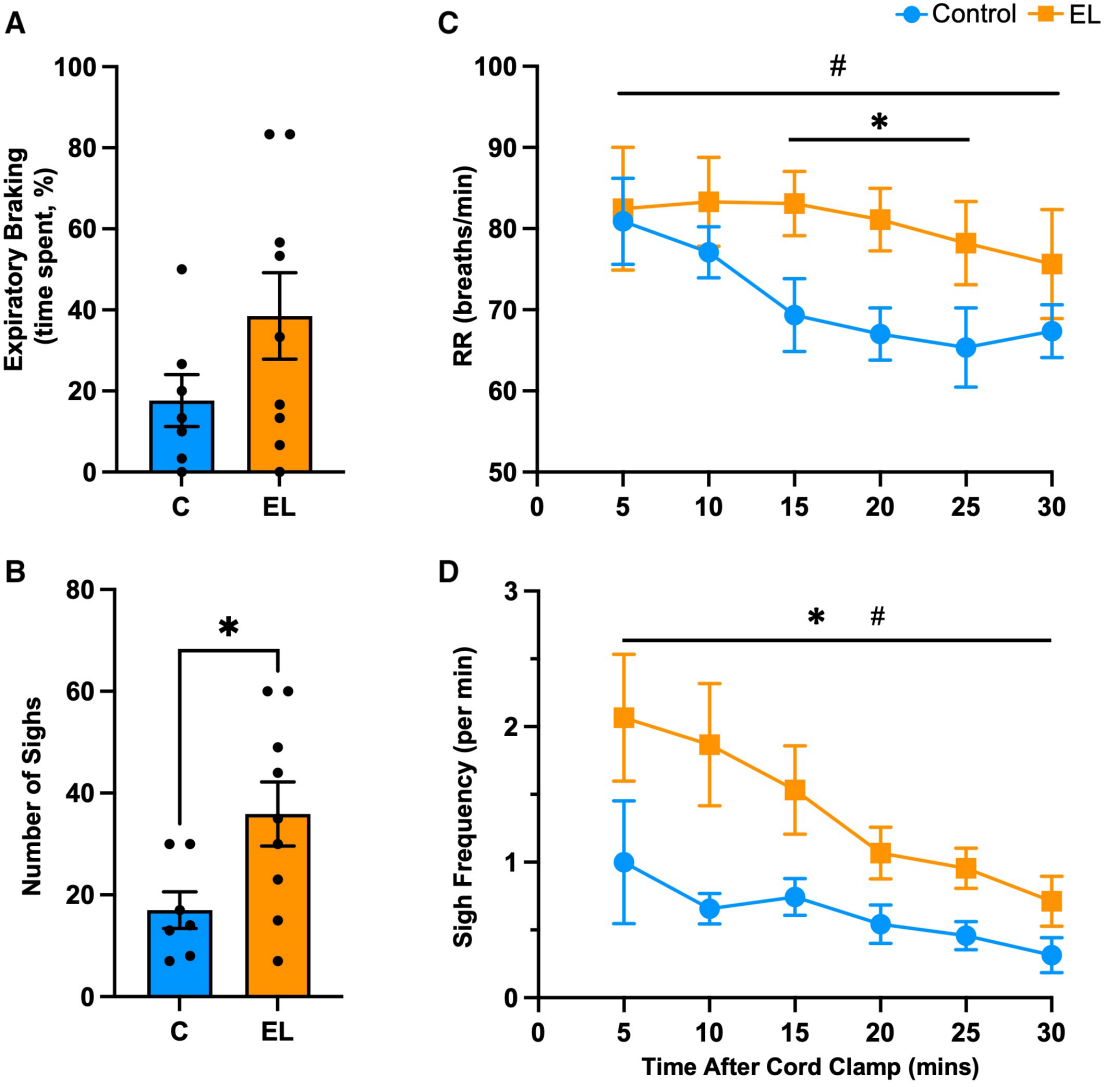
Respiratory behaviour immediately after birth in Control and EL lambs. Proportion of time (%) spent performing expiratory braking manoeuvres (**A**), mean respiratory rates (RR; **C**), total number of sighs (**B**) and sigh frequency (per minute; **D**) within the first 30 minutes after umbilical cord clamping in Control (blue; *n* = 7) and elevated airway liquid (EL; orange; *n* = 10) lambs. Data are mean ± SEM. Unpaired *t*-test (**A**,**B**): *p ≤ 0.05. Mixed effects analysis (**C**,**D**): *p ≤ 0.05 = effect of treatment. ^#^*p* ≤ 0.05 = effect of time.

There was considerable variability in breathing behaviour between lambs in both groups. During the first 30 min after umbilical cord clamping, EL lambs tended to spend more time performing expiratory braking manoeuvres than Controls (38.5 ± 10.7% vs. 17.6 ± 6.4%, *p *= 0.14, Figure 2A). However, despite the fact that expiratory braking necessarily slows respiratory rates, EL lambs had notably higher respiratory rates at 15 to 25 min after cord clamping (*p *= 0.028, Figure 2C). Within 30 min after umbilical cord clamping, EL lambs performed more sighs (deep inspirations) (35.9 ± 6.3 sighs vs. 17.0 ± 3.6 sighs, *p *= 0.031, Figure 2B), with sigh frequency in both groups being highest at 5 min after umbilical cord clamping (Controls = 1.0 ± 0.5 sighs/min, ELs = 2.1 ± 0.5 sighs/min, Figure 2D) before gradually decreasing over the first 30 min (Figure 2D).

### 3.4 Longer term effects of EL on blood gas status and respiratory behaviour

**Figure 3:**
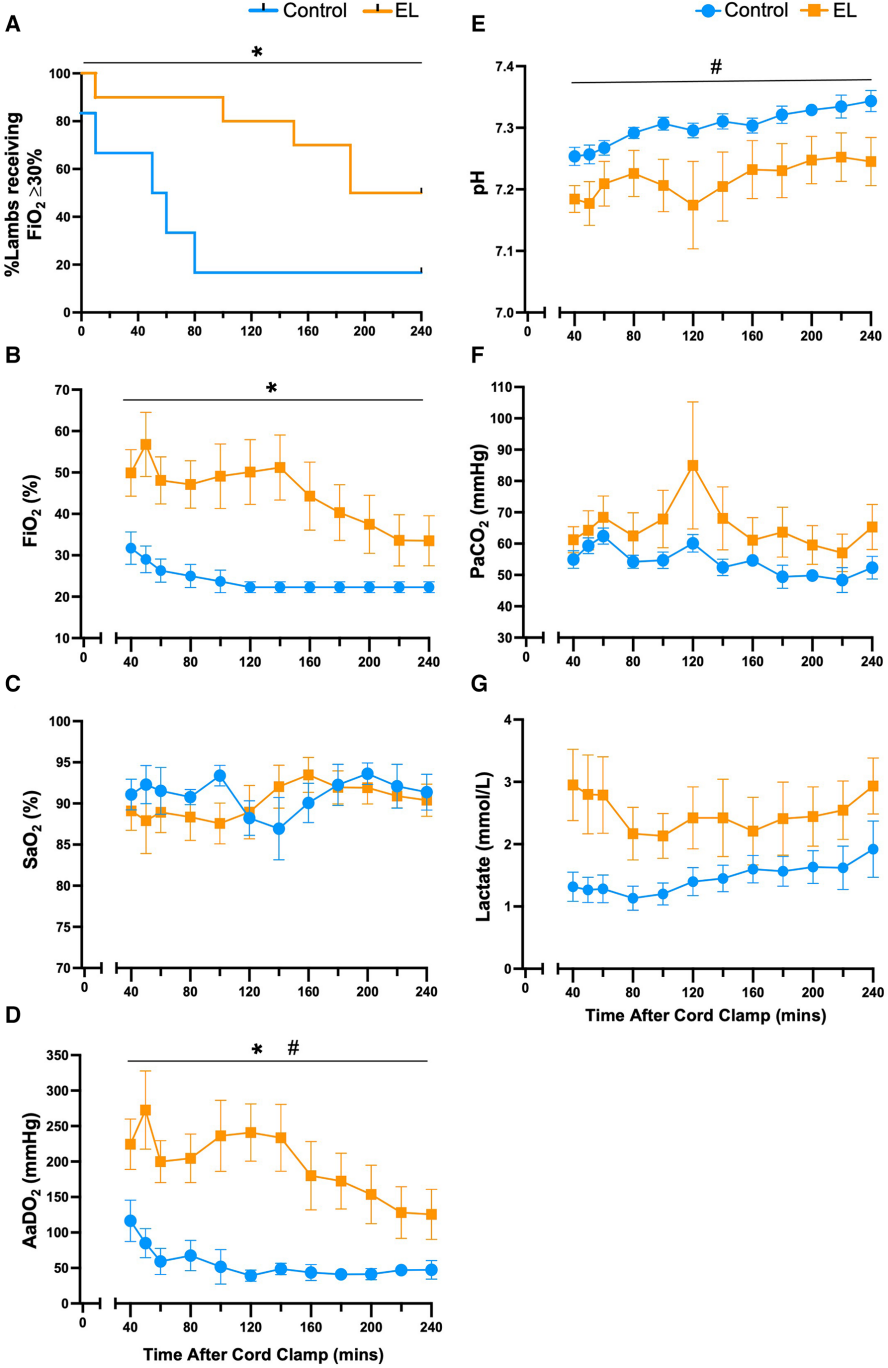
Supplemental oxygen administration, gas exchange capabilities and pH status after birth in control and EL lambs. Proportion of lambs requiring supplemental oxygen (**A**), fraction of inspired oxygen (FiO_2_; **B**), arterial oxygen saturations (SaO_2_; **C**), alveolar-arterial difference in oxygen (AaDO_2_; **D**), arterial pH (**E**), partial pressure of CO_2_ (PaCO_2_; **F**) and lactate (**G**) from 40 min to four hours after umbilical cord clamping in Control (blue circles; *n* = 7) and elevated airway liquid (EL; orange squares; *n* = 10) lambs. Data are mean ± SEM. Log rank test (survival curve; **A**): *p ≤ 0.05. Mixed effects analysis (**B–G**): *p ≤ 0.001, effect of treatment. #*p *≤ 0.05, effect of time.

A greater proportion of Control lambs were able to be weaned off oxygen compared to the EL group (*p *= 0.03, Figure 3A). Oxygen exchange (AaDO_2_) was significantly reduced in EL lambs compared to Controls for at least 4 h after birth (*p *= 0.0002, Figure 3D) and as such EL lambs required significantly more supplemental oxygen (FiO_2_, *p *= 0.003, Figure 3B) to maintain oxygen saturations levels >90% (Figure 3C). On average, Control lambs received 27.5 ± 1.0% FiO_2_ and EL lambs received 49.9 ± 1.5% FiO_2_ (*p *< 0.0001, unpaired *t*-test). While pH improved over time in both Controls and EL lambs, EL lambs tended to have lower pH (*p *= 0.12, Figure 3E) and higher PaCO_2_ levels (*p *= 0.28, Figure 3F) than Controls.

**Figure 4:**
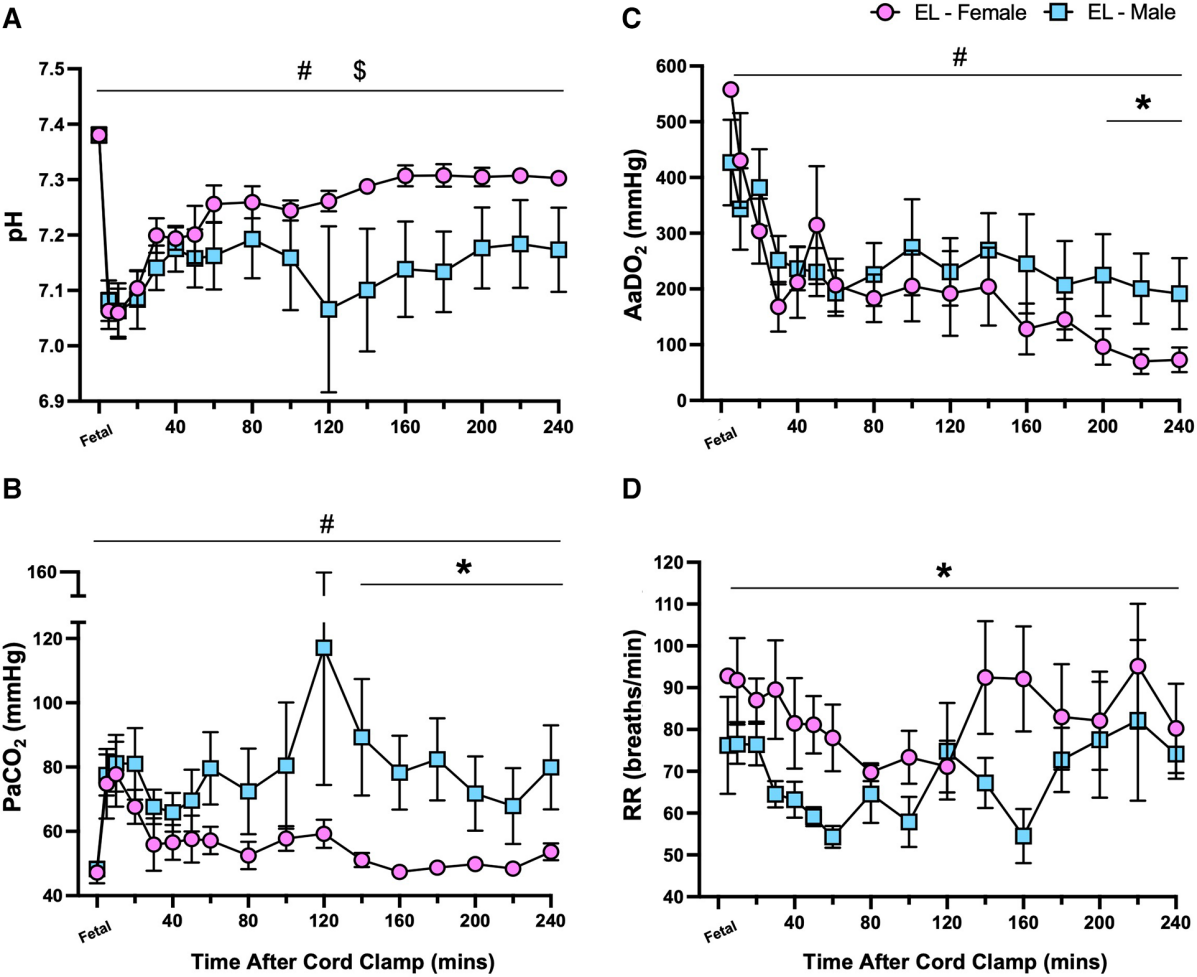
Sex differences in pH status and gas exchange capabilities after birth in lambs with elevated airway liquid. Arterial pH (**A**), partial pressure of CO_2_ (PaCO_2_; **B**), alveolar-arterial difference in oxygen (AaDO_2_; **C**) and respiratory rate (RR; **D**) in the first four hours after umbilical cord clamping in elevated airway liquid female (EL female; pink circles, *n* = 5) and male (EL male; blue squares, *n* = 5) lambs. Data are mean ± SEM. Mixed effects analysis: *p ≤ 0.05, effect of sex. #*p *≤ 0.05, effect of time, $*p *≤ 0.05 = interaction effect (sex × time).

Following transition (>30 min after umbilical cord clamping), major differences between male and female lambs within the EL group were observed. Male EL lambs tended to have lower pH levels than females (*p *= 0.21, Figure 4A), which were driven by higher PaCO_2_ levels in male EL lambs after 140 min (*p *= 0.041, 140–240 min, Figure 4B). Oxygen exchange (indicated by AaDO_2_ levels) was initially similar between EL males and females during the first 3 h, but after 200 min it was significantly higher (indicated by lower AaDO_2_ values) in EL females (*p *= 0.038, Figure 4C). During non-active periods, respiratory rates were significantly higher in EL females than in EL males over the 4 h period (*p *= 0.014, Figure 4D).

**Figure 5:**
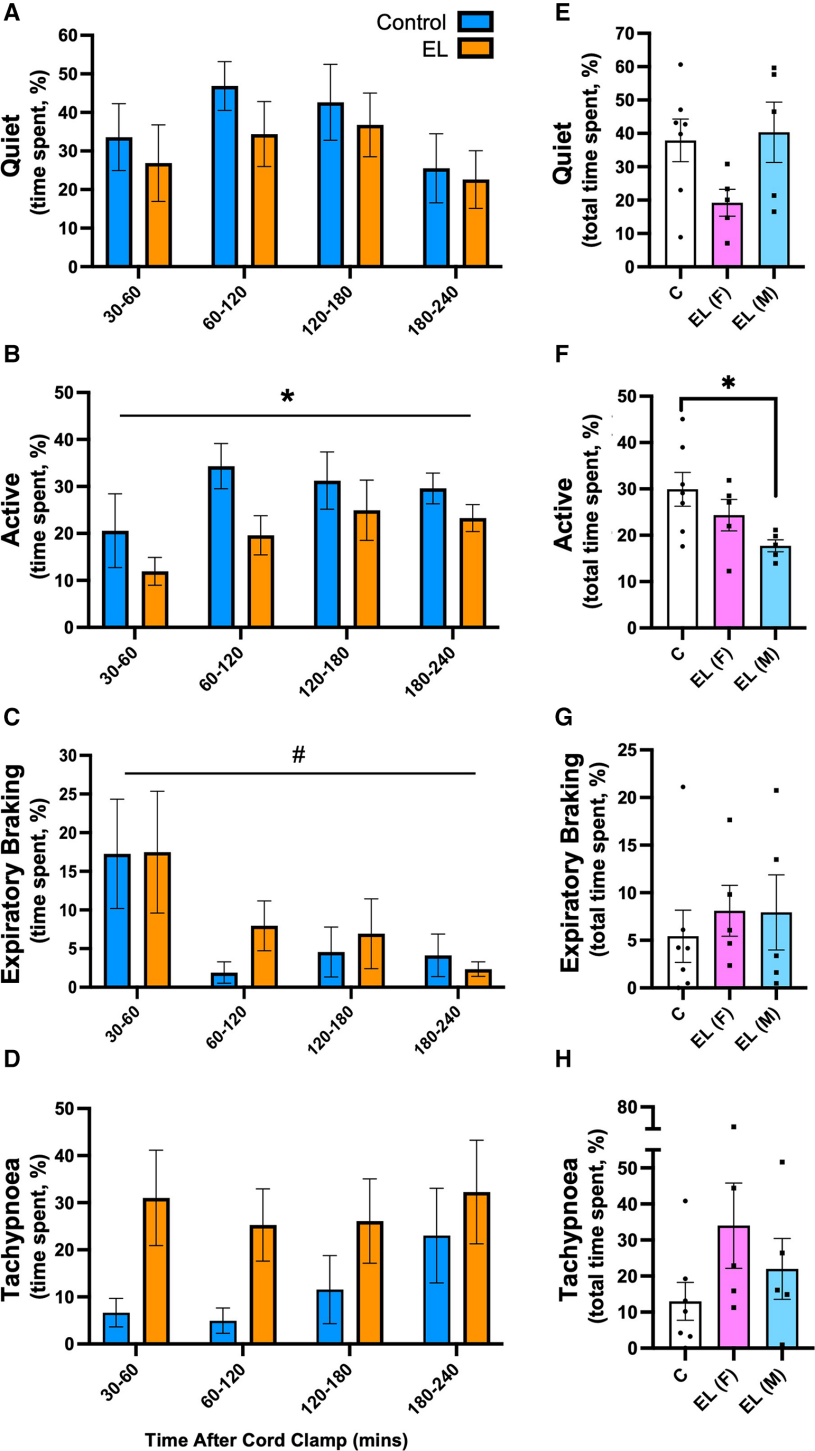
Time spent performing different breathing patterns after birth in Control and EL lambs. Proportion of time (%) spent between 30-60 minutes and in every 60 minute period thereafter following umbilical cord clamping in Quiet (**A**) and Active (**B**), Expiratory Braking (**C**), Tachypnoea (**D**), breathing patterns in Control (blue; *n* = 7) and elevated airway liquid (EL; orange; *n* = 10) lambs. Total cumulative time spent in each breathing pattern (%) is also presented for Control, EL female (F; *n* = 5) and EL male (M; *n* = 5) lambs (**E**–**H**). Data are mean ± SEM. Mixed effects analysis (**A**–**D**): *p ≤ 0.05, effect of treatment. #*p* < 0.01, effect of time. One-way ANOVA (**E**–**H**): *p ≤ 0.05.

In Control lambs, the dominant breathing patterns after birth were quiet breathing and breathing during activity (Figures 5A,B). In EL lambs, the dominant breathing patterns were quiet breathing and tachypnoea (Figures 5A,D). EL lambs were significantly less active than Controls (*p *= 0.049, Figure 5B), which was largely due to lower activity levels in EL males as activity levels in EL females were similar to Controls (*p *= 0.031, Control vs. EL males, Figure 5F).

The incidence of expiratory braking manoeuvres decreased overtime in both Control and EL (males and females) lambs (*p *= 0.033, time effect, Figure 5C). While the incidence of expiratory braking (grunting) was similar between groups (Figure 5G), in Control lambs it most commonly followed feeding. However, as EL lambs spent significantly less time feeding (8.09 ± 1.2% vs. 3.85 ± 1.2%, *p *= 0.031, graph not shown), most of their braking movements were not associated with feeding. One male EL lamb displayed a pattern of consistent diaphragmatic braking, defined as the presence of post-inspiratory diaphragmatic contractions repeating over multiple breaths (see Supplementary Video S1); no Control lambs displayed this breathing pattern.

Tachypnoea was most commonly associated with vigorous physical activity (e.g., kicking legs in attempts to run) in Control lambs, (180–240 min; Figure 5D). In contrast, EL lambs were much less active, but tended to be more tachypneic than Controls (after 30 min), although this was not statistically significant due to high variability between lambs (*p *= 0.093; Figure 5D). Female EL lambs tended to be more tachypneic than Controls and male EL lambs, although this was not significantly different (Control vs. EL female: 12.99 ± 5.3% vs. 33.98 ± 11.8%, *p *= 0.20; EL male vs. EL female: 22.00 ± 8.4% vs. 33.98 ± 11.8%, *p *= 0.61; Figure 5H).

**Figure 6:**
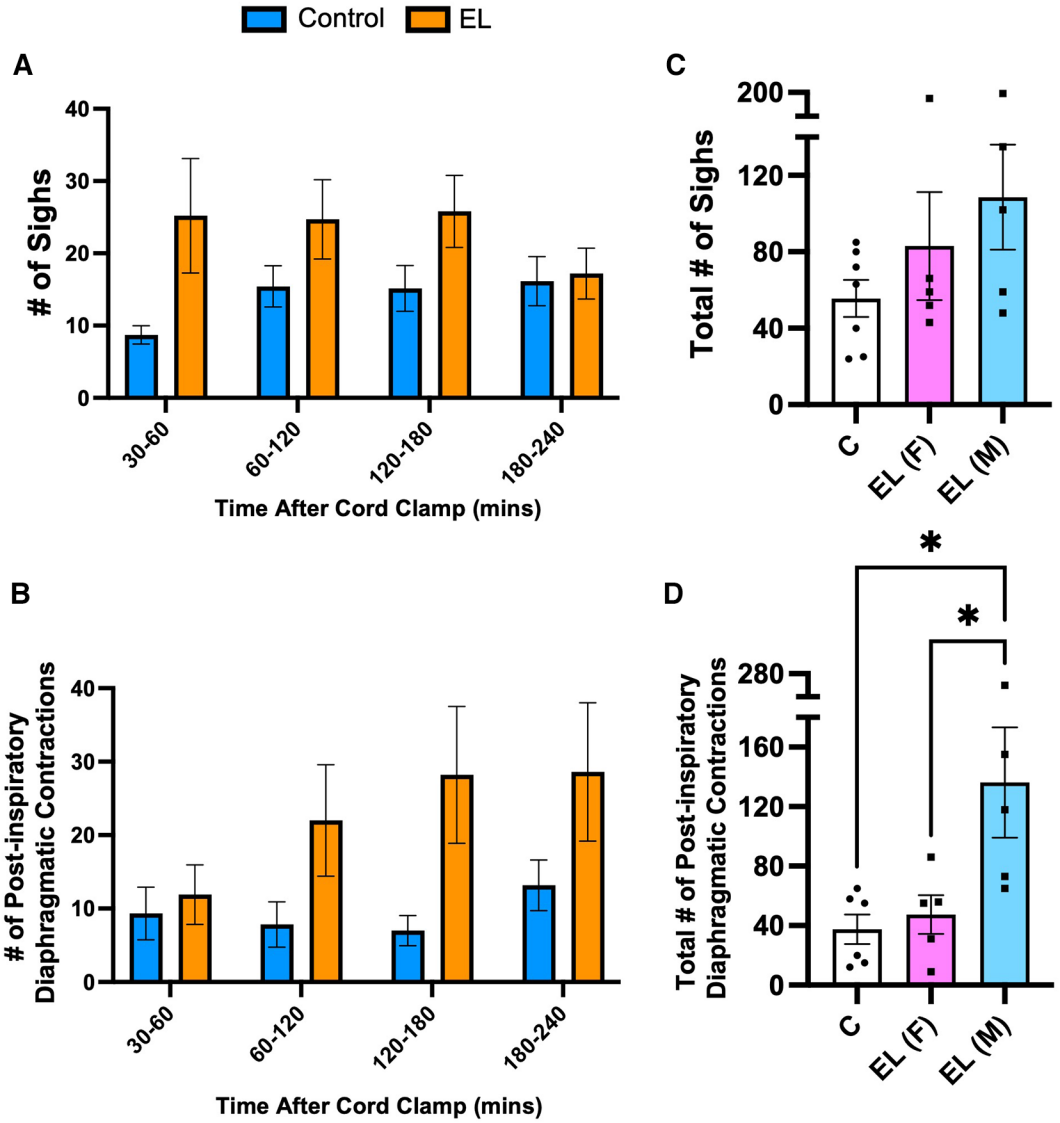
Isolated respiratory features after birth in Control and EL lambs**.** Number of sighs (**A**) and post–inspiratory diaphragmatic contractions (“double breaths”; **B**) were counted from 30–60 minutes and every 60 minute period thereafter following umbilical cord clamping in Control (blue; *n* = 7) and elevated airway liquid (EL; orange; *n* = 10) lambs. Total cumulative number of sighs (**C**) and post–inspiratory diaphragmatic contractions (**D**) are also presented for Control, EL female (F; *n* = 5) and EL male (M; *n* = 5) lambs. Data are mean ± SEM. One–way ANOVA (**C**,**D**): *p ≤ 0.05.

The number of sighs or post-inspiratory diaphragmatic contractions observed after delivery tended to be higher in both male and female EL lambs than Controls (sighs: Control vs. EL female: 56 ± 10 vs. 83 ± 28, *p *= 0.57; Control vs. EL male: 56 ± 10 vs. 109 ± 27, *p *= 0.16, Figure 6C), but over time these differences were not statistically significant (*p* = 0.13 and *p* = 0.11, Figures 6A,B, respectively). Interestingly, EL males showed significantly more post-inspiratory diaphragm contractions compared to both Control lambs and EL females (Control vs. EL male: 38 ± 10 events vs. 136 ± 37 events, *p *= 0.018; EL female vs. EL male: 47 ± 13 events vs. 136 ± 37 events, *p *= 0.041, Figure 6D). Similar to expiratory grunting, post-inspiratory diaphragmatic contractions commonly occurred after feeding periods.

### 3.5 Effect of EL on relative respiratory morbidity after birth

**Figure 7:**
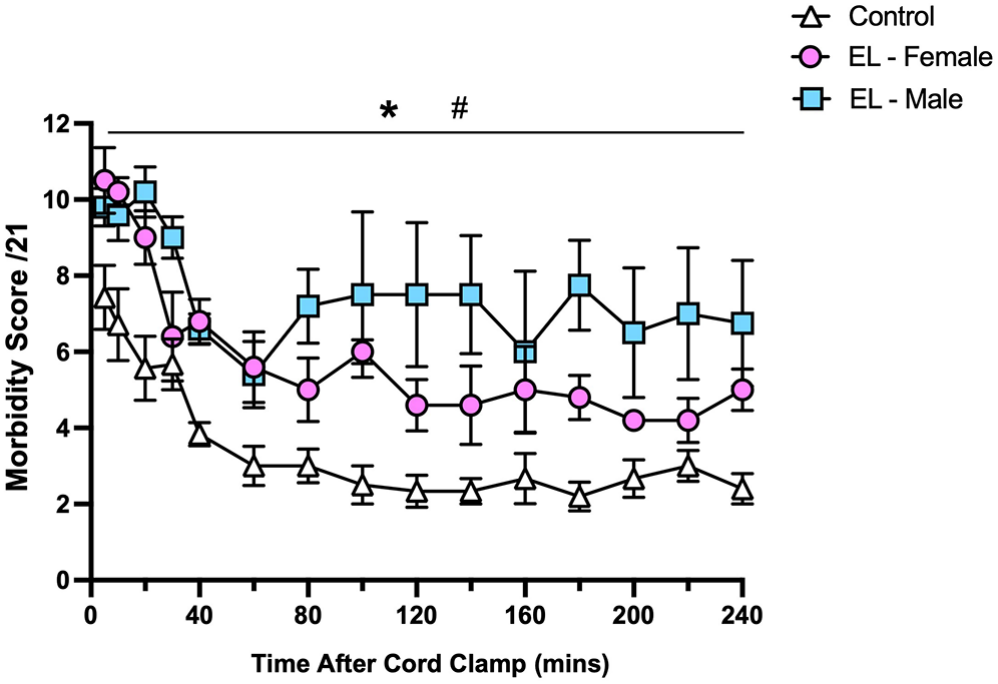
Relative respiratory morbidity during the first four hours after birth in control and EL lambs. Respiratory morbidity was scored (out of 21) using the criteria outlined in Table 1. Scores in Control (white triangles; *n* = 7), elevated liquid female (EL—Female; pink circles; *n* = 5) and elevated liquid male (EL—Male; blue squares; *n* = 5) lambs in the first four hours after umbilical cord clamping are presented. Data are mean ± SEM. Mixed effects analysis: *p < 0.0001, effect of treatment. #*p *< 0.0001, effect of time.

Morbidity scores were highest in the immediate newborn period during stabilisation in Control and EL lambs and then significantly decreased over the first hour after birth before remaining relatively stable over 3 h (*p *< 0.0001, time effect, Figure 7). While both EL males and EL females maintained higher morbidity scores than Controls (*p *< 0.0001, Figure 7), EL males tended to have higher morbidity scores than EL females, although this did not reach statistical significance (*p *= 0.094).

### 3.6 Predicting gas exchange capabilities in male and female lambs after birth

Multiple linear regression modelling was used to identify respiratory features (expiratory braking, respiratory rate, isolated respiratory events such as sighs and post-inspiratory diaphragmatic contractions) and other physiological measures that best predict gas exchange capabilities (AaDO_2_) in male (Table 3) and female lambs (Table 4) after birth. Variables that were positively correlated with AaDO_2_ (presence of elevated airway liquid at birth, expiratory braking, respiratory rate and frequency of post-inspiratory diaphragmatic contractions) were highly significant predictors of poorer gas exchange in male lambs (Table 3), with frequency of post-inspiratory diaphragmatic contractions being the most significant predictor of poor gas exchange (*p *< 0.0001). Time after umbilical cord clamping, pH status at 5 min after umbilical cord clamping and sigh frequency were not significant predictors of AaDO_2_ in male lambs (Table 3). An alternative regression model run in female lambs only, indicated that time after birth and presence of elevated airway liquid were the only significant predictors of AaDO_2_ (*p *< 0.0001, Table 4). As we anticipated that EL would result in a mild form of RD in these lambs, and that EL females exhibited relatively lesser respiratory morbidity, it is likely we are underpowered to detect significant predictors of poor gas exchange in female lambs.

**Table 3:** Multiple linear regression model for predicting gas exchange (AaDO_2_) in near-term male newborn lambs.

Variable	Estimate	Standard error	95% CI	*P* value
Intercept	−35.75	30.55	−95.91 to 24.42	0.243
Time after umbilical cord clamping (ref = no)	−0.123	0.098	−0.32 to 0.07	0.210
Elevated airway liquid at birth (ref = no)	163.8	17.93	128.5 to 199.1	**<0**.**0001**
pH <7.2 at 5 min after umbilical cord clamping (ref = no)	6.94	20.87	−34.17 to 48.04	0.74
Expiratory braking (ref = no)	42.76	16.56	10.15 to 75.37	**0**.**010**
Respiratory rate (breaths/min)	0.971	0.374	0.23 to 1.71	**0**.**010**
Sighs (*f*)	14.25	12.00	−9.39 to 37.89	0.240
Post-inspiratory diaphragmatic contractions (*f*)	46.82	8.31	30.46 to 63.18	**<0**.**0001**

Goodness of fit: *R*^2^ = 0.5428, Adjusted *R*^2^ = 0.5301, AICc = 2360.

The bold values indicate the variable passes the threshold for statistical significance.

**Table 4:** Multiple linear regression model for predicting gas exchange (AaDO_2_) in near-term female newborn lambs.

Variable	Estimate	Standard error	95% CI	*P* value
Intercept	150.2	22.22	106.5 to 194.0	**<0**.**0001**
Time after umbilical cord clamping (ref = no)	−0.702	0.078	−0.855 to −0.548	**<0**.**0001**
Elevated airway liquid at birth (ref = no)	90.38	12.32	66.14 to 114.6	**<0**.**0001**
pH <7.2 at 5 min after umbilical cord clamping (ref = no)	31.75	16.23	−0.189 to 63.68	0.051
Respiratory rate (breaths/min)	−0.194	0.275	−0.734 to 0.346	0.480
Post-inspiratory diaphragmatic contractions (*f*)	27.68	16.25	−4.29 to 59.64	0.089

Goodness of fit: *R*^2^ = 0.3685, Adjusted *R*^2^ = 0.3586, AICc = 2902.

The bold values indicate the variable passes the threshold for statistical significance.

## 4 Discussion

The volumes of liquid present in the airways at birth in newborns delivered by elective CS without labour are similar to a late gestation fetus (8) and are considerably greater than the volume expected in infants delivered vaginally (26). While it is known that greater airway liquid volumes adversely impact on cardiorespiratory function, we have examined how these greater volumes affect breathing patterns in spontaneously breathing lambs. We found that greater liquid volumes reduced gas exchange, as indicated by a lower pH, high PaCO_2_ and a greater requirement for supplemental oxygen, and altered respiratory behaviour after birth. These effects of EL were more pronounced in males compared to female lambs. EL lambs were less active, fed less and had higher levels of respiratory morbidity that included a mixture of tachypnoea, expiratory braking, intercostal retractions, nasal flaring, sighs and “double breaths”. These findings demonstrate that EL volumes at birth induce respiratory behaviours in spontaneously breathing lambs that are commonly associated with RD in term/near-term infants. However, the pattern of behaviours was highly variable and complex and changed with time.

### 4.1 RD in term and near-term newborns

RD in term or near-term newborns exists as a spectrum of severity, most probably because the volume of liquid present in the airways at birth varies hugely between infants (8). It is not surprising, therefore, that there is considerable uncertainty as to what is normal respiratory behaviour, how these behaviours change to reflect RD and how infants with RD should be managed after birth to avoid the development of severe lung disease. Near-term and term newborns are not expected to develop RD and the diagnosis is subjective and often made well after symptoms have resolved or have manifested to a level that requires treatment. In view of our findings, this is understandable as there is no one specific behaviour that differentiates RD from normal respiratory behaviour. Instead, it is a combination of many behaviours that best define RD in near-term newborns. For instance, in Control lambs, tachypnoea was relatively common, but this was usually associated with physical activity, and while grunting was not uncommon immediately after birth, it mostly only occurred after feeding in later hours. Thus, individually these behaviours are not indicative of RD. Instead, we suggest that the early signs of RD could include newborns that are less active, less inclined to feed, are mildly hypoxic, have tachypnoea that is not associated with activity and display expiratory braking as well as an increased incidence of sighs. Studies in The Netherlands involving pulse oximetry screening for congenital heart disease have demonstrated that infants with RD (wet lung) can indeed be detected early (27). While mild hypoxia was the primary reason why these infants were admitted to the NICU, respiratory symptoms did not manifest until they were in the unit and closely monitored (27). Furthermore, if newborns exhibit post-inspiratory diaphragmatic contractions (i.e., double breaths) that are not associated with feeding, our data suggest that this is a highly significant sign of more severe RD.

While it has long been assumed that the primary cause of RD in term and near-term infants is a reduced ability to clear airway liquid after birth, imaging studies have shown that breathing activity (specifically inspiration) is responsible for >95% of airway clearance after birth (11). As the presence/absence of breathing is not the issue, the cause of the RD cannot be due to a failure of the mechanism responsible for airway liquid clearance. Instead, recent evidence indicates that larger airway liquid volumes at birth are responsible, which increases the degree of pulmonary oedema in the first few hours after birth. We calculate that our EL lambs had ∼30 ml/kg of liquid in their airways at birth and so our model likely simulates only a mild form of RD (fetal airway liquid levels at term can be as high as 35–40 ml/kg). Nevertheless, some of our EL lambs (males in particular) displayed a significant degree of RD and large reductions in gas exchange, which confirms that greater than expected liquid volumes at birth induces respiratory behaviour and breathing patterns that resemble those observed clinically in term and near-term infants with RD.

### 4.2 Time related changes in breathing patterns and respiratory function

Infants born with higher airway liquid volumes face two distinct challenges to their respiratory function after birth (7): (i) the clearance of larger liquid volumes into peri-alveolar interstitial tissue to establish gas exchange (i.e., short term challenge) and (ii) the accommodation of more liquid within the lung's interstitial tissue compartment (i.e., long term challenge). The long-term challenge involves development of pulmonary oedema, which increases interstitial pressures and necessitates expansion of the chest wall to ensure that gas can remain in the airways at end-expiration (7). Both of these challenges likely impact on respiratory behaviour in the immediate newborn period. Initially, larger or more inspiratory efforts are required to generate the transpulmonary pressures needed to drive liquid through the airways and across the distal airway wall. While we were unable to measure lung aeration in this study and relate it to inspiratory activity, this relationship has been shown in mechanically ventilated newborn rabbits with EL. Specifically, these EL newborns required almost twice as many inflations to move airway liquid across the lung over a longer time period to achieve the same level of aeration as a newborn with less airway liquid present at birth (i.e., the volume expected after a vaginal delivery) (13). In spontaneously breathing EL lambs, we observed a significant increase in sighs or deep inspirations soon after birth that likely reflects a recurring need to either contribute to airway liquid clearance or oppose airway liquid reflooding between breaths, which is common in newborns with EL (13, 15).

To overcome the long-term challenge of EL, newborns must actively defend their newly formed functional residual capacity (FRC) against airway liquid reflooding and the reduction in lung compliance caused by pulmonary oedema (28). FRC can be defended by employing expiratory braking manoeuvres to interrupt and reduce expiratory gas flows and lengthen the expiratory phase of the respiratory cycle. This is achieved by partial or total adduction of the glottis that preserves lung volume during expiration, coupled with active recruitment of expiratory muscles to complete expiration before the next inspiration. While we observed these expiratory braking/grunting behaviours in both Control and EL lambs, particularly immediately after birth, they were more common in EL lambs. Furthermore, when grunting was observed in Control lambs at >30 min after birth, it commonly followed feeding and was likely due to a full stomach pushing up on the diaphragm. While this will reduce FRC and thereby triggering braking and grunting, this is clearly a normal physiological process that is not indicative of RD. Similarly, post-inspiratory diaphragmatic contractions also occurred in Controls following feeding periods and may contribute to FRC preservation. Nevertheless, by pressurising the airways and slowing the rate of lung deflation, these expiratory braking manoeuvres help to protect against FRC loss and if followed by a deep inspiratory effort, any liquid that has re-entered the airways will be re-cleared (11).

Large degrees of pulmonary oedema at birth also necessarily increases the degree of chest wall expansion that is required to sustain any given FRC after birth (13). This limits the newborn's inspiratory reserve capacity and likely contributes to an increased work of breathing due to a higher recoil pressure associated with greater chest wall expansion. As this must increase the energy cost of breathing, newborns with EL may be more vulnerable to fatigue, which may have contributed to the lack of activity and a reduced desire to feed in EL compared to Control lambs.

During lung aeration, pulmonary interstitial tissue pressures initially increase as liquid moves into lung tissue (first 3 h after birth) and then gradually decrease over the subsequent hours (3–6 h after birth) to eventually become sub-atmospheric (29). This gradual decrease in pressure likely reflects resolution of the pulmonary oedema as the liquid is cleared from lung tissue. Interestingly, this time course is similar to the time course for the onset and then self-resolution of mild RD in term and near-term human infants. We found that the liquid content of lung tissue (wet/dry lung weight ratios) was similar in Control and EL lambs at 4 h after birth, which is consistent with the expectation that most of the excess liquid will have been cleared from lung tissue by this time. As this liquid should be excreted from the body as urine, this is also consistent with our finding that urine production rates tended to be greater in EL lambs, despite having a markedly lower milk intake than Controls. The latter finding likely explains why urine production rates were not statistically significant between EL and Control lambs and further indicates that our model only reflects a mild version of RD.

While the incidence of braking was highest in EL lambs over the first 30 min after birth, this incidence markedly reduced over time and by 1–2 h after birth was not different to Controls. This time course suggests that the braking was due to a direct effect of liquid within lung tissue either by affecting FRC levels or by activating pulmonary oedema receptors (J receptors). However, the time course for the changes in tachypnoea was considerably more complex, probably due to multiple interacting factors. In EL lambs, the highest rates of tachypnoea (when activity was at its lowest) occurred during the first 60 min after birth, which could be due to J-receptor activation, as occurs in adults (30). However, if this was the sole mechanism, we would expect the incidence of tachypnoea to decrease with time as the liquid is cleared from lung tissue, but it remained high in EL lambs. However, it is possible that the higher tachypnoea rates were largely due to female EL lambs who may exhibit a greater sensitivity to physiological factors that stimulate breathing (e.g., CO_2_); see below.

### 4.3 Breathing patterns that characterise RD

As EL lambs displayed a wide variability of breathing behaviours at different times after birth, there was no “classic” breathing pattern that could be used to categorically define this form of RD. Instead, it is likely to be a combination of behaviours including; (i) tachypnoea without activity, (ii) expiratory braking and grunting with clear signs of laboured breathing (intercostal retractions and nasal flaring) not associated with feeding, (iii) reduced levels of activity and feeding and (iv) mild hypoxia within 30–60 min of birth. It is interesting that periods of expiratory braking (particularly in male lambs) were commonly associated with hypercapnia, which was likely due to lower minute ventilation rates caused by the braking and/or to higher energy consumption associated with an increased work of breathing. Nevertheless, the preference for male lambs to sustain expiratory braking patterns despite high CO_2_ levels (>80 mmHg), suggests that the afferent signals responsible for activating expiratory braking (i.e., oedema and stretch receptors) can override the sensory input from peripheral and central chemoreceptors that would normally increase respiratory rates in response to higher CO_2_ levels. This could also indicate that males have a higher chemosensitivity threshold and so are less sensitive to higher CO_2_ levels than females, or that females initially respond to pulmonary oedema to a greater extent than males. Furthermore, as most lambs with tachypnoea (up to 200 breaths/min) were normocapnic, CO_2_ levels did not appear to be a major driver of tachypnoea development in males or females, particularly after the first hour.

Another interesting respiratory pattern we observed in EL lambs were continuous post-inspiratory diaphragmatic contractions or “diaphragmatic braking” which, in combination with hypercapnia, are features that have been previously observed in preterm infants (31). Diaphragmatic braking was most likely to occur in male EL lambs from 2 to 4 h after birth when they were markedly hypercapnic (PaCO_2_ > 80 mmHg; see Supplementary Video S1). This braking manoeuvre was observed as either regular isolated post-inspiratory diaphragmatic contractions (∼1–2 events/min), or periods of sequential diaphragmatic braking lasting many breaths. Often, the second post-inspiratory diaphragmatic contraction led to a greater reduction in intrathoracic pressure, suggesting that the glottis may have relaxed and was not actively abducted during the second contraction, as would normally occur during inspiration. We have recently observed this manoeuvre in newborn rabbits using phase contrast x-ray imaging (unpublished observations). Clearly, there is a complex interplay of both mechanical and ventilatory factors that may contribute to alterations in breathing patterns after birth, which is compounded by the degree of pulmonary oedema/presence of elevated airway liquid.

### 4.4 Study limitations and future directions

Unlike CS performed in humans, ethical requirements for this experiment mandated that we use maternally administered sedatives during both the induction of spinal anaesthesia (propofol) and during the CS (midazolam). As both propofol and midazolam can cross the placenta and may cause respiratory depression in the fetus/newborn lamb, exposure to these drugs may have had an impact on breathing patterns observed soon after birth. While we aimed to reduce the impact of maternally administered sedatives by using relevant antagonists, in addition to using respiratory stimulants (caffeine and oxygen), these drugs are not routinely used in near-term/term infants at birth.

While we did not initially power this study to detect sex differences as a primary outcome, we were able to detect a significant difference in a number of physiological measures between males and females within the EL group (i.e., PaCO_2_, AaDO_2_, average respiratory rates, %time spent active and frequency of post-inspiratory diaphragmatic contractions). We are, however, likely to be underpowered to detect differences related to sex in some of the other measures such as %time spent quiet, tachypneic and expiratory braking or frequency of sighs. Regardless, we believe this study provides an indication that males and females with RD may present differently clinically and further investigation into the effect of sex on the response to pulmonary oedema after birth is warranted. This study can provide the basis for more appropriately powered pre-clinical studies conducted in the future.

## 5 Conclusion

We have shown that elevated airway liquid volumes at birth alter breathing patterns and respiratory behaviour in spontaneously breathing near-term newborn lambs that are analogous to those displayed by near-term infants with RD. While there are no set breathing patterns and/or behaviours that can be used to define RD in term infants, our observations can be used to develop a clinical score that likely provides the best opportunity to diagnose affected infants early before the symptoms manifest into severe respiratory morbidity. With an increased physiological understanding, the next challenge is to develop more effective management strategies, but this will rely heavily on early identification of the developing RD.

## Data Availability

The raw data supporting the conclusions of this article will be made available by the authors, without undue reservation.

## Supplementary material

Supplementary Video 1 Diaphragmatic braking in a male lamb that developed respiratory distress after birth. A male elevated liquid (EL) lamb in respiratory distress displayed diaphragmatic braking, defined as repeating breaths that included a post-inspiratory diaphragmatic contraction (seen as ‘double breaths’). This lamb also displayed intercostal retractions and nasal flaring. Video was taken approximately 140 minutes after umbilical cord clamping. Lamb was acidotic (pH = 7.084, PaCO_2_ = 103 mmHg, Lactate = 1.1 mmol/L), with arterial oxygen saturations of 87.7% whilst receiving supplemental oxygen (FiO_2_ 70%, 5 L/min) via nasal cannula.
